# Ethnic Differences in Glycaemic Control in People with Type 2 Diabetes Mellitus Living in Scotland

**DOI:** 10.1371/journal.pone.0083292

**Published:** 2013-12-16

**Authors:** Preeti H. Negandhi, Nazim Ghouri, Helen M. Colhoun, Colin M. Fischbacher, Robert S. Lindsay, John A. McKnight, John Petrie, Sam Philip, Naveed Sattar, Sarah H. Wild, on behalf of the Scottish Diabetes Research Network Epidemiology Group

**Affiliations:** 1 Indian Institute of Public Health, Public Health Foundation of India, Gurgaon, Haryana, India; 2 Institute of Cardiovascular and Medical Sciences, BHF Glasgow Cardiovascular Research Centre, University of Glasgow, Glasgow, Scotland, United Kingdom; 3 Biomedical Research Institute, Mackenzie Building, University of Dundee, Dundee, Scotland, United Kingdom; 4 Information Services Division, NHS National Services Scotland, Edinburgh, Scotland, United Kingdom; 5 Metabolic Unit, Western General Hospital, Edinburgh, Scotland, United Kingdom; 6 Department of Diabetes and Endocrinology, Aberdeen Royal Infirmary, Aberdeen, Scotland, United Kingdom; 7 Centre for Population Health Sciences, University of Edinburgh, Medical School, Edinburgh, Scotland, United Kingdom; University of Verona, Ospedale Civile Maggiore, Italy

## Abstract

**Background and Aims:**

Previous studies have investigated the association between ethnicity and processes of care and intermediate outcomes of diabetes, but there are limited population-based studies available. The aim of this study was to use population-based data to investigate the relationships between ethnicity and glycaemic control in men and women with diabetes mellitus living in Scotland

**Methods:**

We used a 2008 extract from the population-based national electronic diabetes database of Scotland. The association between ethnicity with mean glycaemic control in type 2 diabetes mellitus was examined in a retrospective cohort study, including adjustment for a number of variables including age, sex, socioeconomic status, body mass index (BMI), prescribed treatment and duration of diabetes.

**Results:**

Complete data for analyses were available for 56,333 White Scottish adults, 2,535 Pakistanis, 857 Indians, 427 Chinese and 223 African-Caribbeans. All other ethnic groups had significantly (p<0.05) greater proportions of people with suboptimal glycaemic control (HbA1c >58 mmol/mol, 7.5%) compared to the White Scottish group, despite generally younger mean age and lower BMI. Fully adjusted odds ratios for suboptimal glycaemic control were significantly higher among Pakistanis and Indians (1.85, 95% CI: 1.68–2.04, and 1.62,95% CI: 1.38–1.89) respectively.

**Conclusions:**

Pakistanis and Indians with type 2 diabetes mellitus were more likely to have suboptimal glycaemic control than the white Scottish population. Further research on health services and self-management are needed to understand the association between ethnicity and glycaemic control to address ethnic disparities in glycaemic control.

## Introduction

Worldwide the prevalence of diabetes mellitus (DM) is increasing, with increases expected to be greater than previously projected [1], [2], predominantly due to the growing incidence of type 2 diabetes mellitus (type 2 DM) [3]. Primary care data from the UK indicate that the prevalence of DM was 4.26% in 2010, with the prevalence in Scotland estimated at 4.6% [4]. Scotland has an established minority ethnic population, representing an estimated 2% of Scotland's population in 2001 [5]. Although 2011 census data by ethnicity are not yet available it is anticipated that this proportion has increased as it has in the rest of the UK. The largest minority ethnic group within Scotland are South Asians - people of Indian, Pakistani, Bangladeshi and Sri Lankan origin, making up 55% of Scotland's minority ethnic population; with people of Chinese origin making up around 17% of Scotland's minority ethnic population in 2001.

It is recognized that ethnicity influences risk of DM (specifically type 2 DM), globally and within the UK [1], [6]. The prevalence of type 2 DM in migrant South Asians living in the UK and USA is up to five times higher than that of their respective indigenous populations [6]. Further, the age of onset of type 2 DM in South Asians appears to be around 10 years earlier and occurs at a lower body mass index (BMI) than among populations of European ancestry [7].

Recent data on disease management and natural history of DM in minority ethnic groups, in particular South Asians with type 2 DM in the UK indicate persisting differences [8], related to suboptimal processes of care and differences in intermediate outcomes [7]–[11], [11,12]. One study showed that South Asians had greater mean deterioration in haemoglobin A1c (HbA1c) than Europeans after 5-years of follow-up [7]. Another study showed that from 2004-9 whilst the proportion of patients with HbA1c <58 mmol/mol, (<7.5%) increased over time in white Europeans, South Asians and African-Caribbeans living in London, only 48% of South Asians had optimal control (HbA1c <58 mmol/mol, <7.5%) compared to 56% of white Europeans [12]. Another study showed that although South Asians with type 2 DM were 1.11 times (95% CI 1.06, 1.16) more likely to have a structured review, their HbA1c levels were 1.03 times higher (95% CI 1.00, 1.06) and retinopathy was 1.36 times more common (95% CI 1.03, 1.78) than the non-South Asians [8]. Finally despite introduction of a pay for performance incentive in U.K. primary care being associated with improvements in the intermediate outcomes of diabetes care for all ethnic groups, between 2000 and 2005 the magnitude of improvement relative to predicted improvement was higher for white Europeans compared to blacks or South Asians living in London.

The majority of large-scale published UK data on ethnicity and diabetes focuses on South Asians with DM living in England, with little data from elsewhere in the UK. Scotland has developed and maintained a national electronic dataset for people with diagnosed diabetes that covers approximately 99.5% of the population of 5 million people that is used for clinical care and includes data on potential confounding factors [13]. The aim of this study was to use these population-based data to investigate the relationships between ethnicity and glycaemic control in men and women with type 2 DM living in Scotland, with and without adjustment for potential confounding factors.

## Methods

### Study population and data extraction

Population-based data are available for people with diagnosed diabetes in Scotland from the Scottish Care Information–Diabetes Collaboration (SCI-DC) dataset [13]. Briefly, the dataset has existed at a national level since 2000, contains demographic and clinical data relevant to diabetes care that is updated on a daily basis from approximately 995 of the 1,000 GP practices in and secondary care databases across Scotland. Further details of the dataset are available from previous papers [13], [14]. People with diabetes are asked to identify their ethnic group from a standard list used in the 2001 Census in Scotland

For the present retrospective cohort study, anonymised data were extracted in May 2008. The dataset initially contained records of 198,622 patients with diabetes. This analysis included people of White Scottish, Indian, Pakistani, Chinese and African-Caribbean ethnic groups. People of Bangladeshi origin were excluded due to small numbers. Optimal glycaemic control among people with diabetes relevant to the period of analysis was considered as an HbA1c <58 mmol/mol (7.5%) [15], using the mean of all HbA1c results available for each patient up to May 2008 in the database. Socio-economic status (SES) categorised in to fifths, with the lowest fifth representing the most affluent, and highest fifth representing the most deprived group, was assigned using an area-based measure based on postcode of residence and the Scottish Index of Multiple Deprivation 2006 score (see http://www.scotland.gov.uk/Topics/Statistics/SIMD for more information).

**Figure 1:**
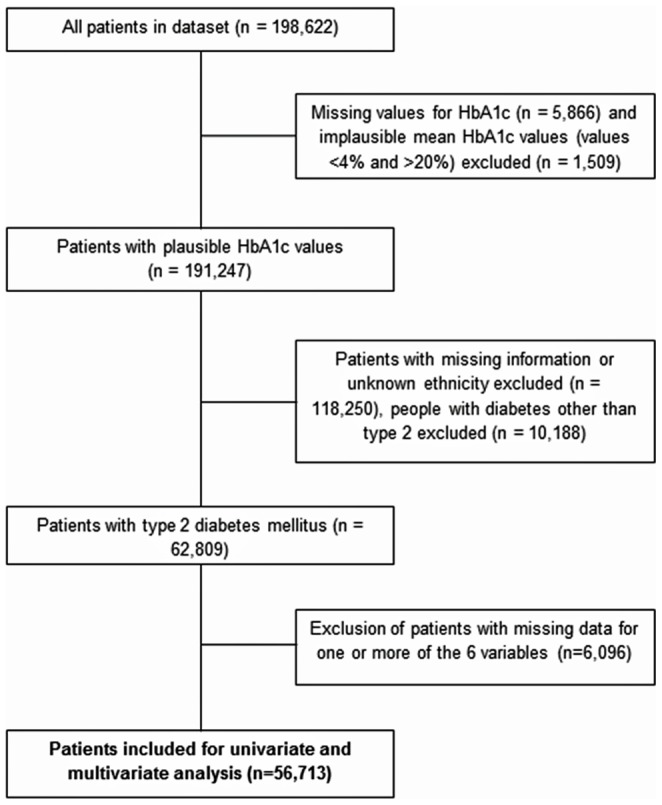
Flow chart showing inclusion and exclusion of cases for analysis.

The study population was determined by excluding subjects who had implausible data, a diagnosis of diabetes other than type 2 DM and incomplete data (Figure 1). Type of diabetes was established using an algorithm that included clinical record of type of diabetes, age at onset and use of insulin/oral hypoglycaemic agents. In univariate analyses, each potential confounding variable (sex, age, SES, mean BMI, prescribed treatment, duration) was individually compared against the exposure (ethnicity) and outcome (mean glycaemic control following entry into the database) to investigate possible associations. The association between ethnicity and glycaemic control was further investigated using logistic regression. Variables were adjusted for in four stages, adjusting initially for only age and sex, then adding SES; BMI and finally all variables. Implausible values for the variables were excluded before conducting the analyses.

### Data analysis

All analyses were performed using categorical variables and chi-square analysis was performed when comparing data between ethnic groups (with the exception of HbA1c values which were presented as continuous variables and Wilcoxon testing was compared when comparing data between ethnic groups). The age of the patients in 2008 was converted into a categorical variable, with four major age-groups formed -≤30 years, 31–50 years, 51–70 years and >70 years. BMI values (mean of all available values on database) were categorized into five categories according to the WHO system of classification for people of European extraction- <18.5 kg/m^2^ (low), 18.5–24.99 kg/m^2^ (normal), 25–29.99 kg/m^2^ (overweight), 30–39.99 kg/m^2^ (obese) and ≥40 kg/m^2^ (morbidly obese). Treatment related variables were combined into one variable on the basis of whether the patients were prescribed insulin or oral drugs, both or no/unrecorded data. Duration of diabetes (in years) was categorized into 5 groups -≤1, 2–5, 6–10, 11–15 and >15 years.HbA1c values were converted into binary variables, using the overall mean of all HbA1c measurements available for each patient and the most recently measured HbA1c value. In addition to the <58 mmol/mol (7.5%) cut-off, further analyses were performed with data categorized using 53 mmol/mol (7%) as a cut-off to define optimal glycaemic control.

All analyses were conducted using SPSS 17. Regression analyses were conducted using the backward stepwise method of inclusion/exclusion of variables in the model. The level for inclusion of the adjusted variables was specified as p≤0.05. For each variable, the reference category was the one with the largest proportion of people and all other categories were compared against this group. The association was summarized as p values, odds ratio (OR) and adjusted odds ratio (AOR) with 95% CI.

### Ethical approval

Approval to generate the pseudonymised research dataset without requiring individual patient consent was obtained from the "Scotland A" multi-centre research ethics committee, Caldicott guardians and the Privacy Advisory Committee of NHS National Services Scotland.

## Results

### Demographic and clinical data

Data for people with implausible/missing values for HbA1c were excluded from the analyses (n = 7,375) as shown in Figure 1. Of the 191,247 people with plausible HbA1c values, 62,809 had type 2 diabetes and ethnicity data recorded. From this cohort, 60,375 people from one of the five ethnicities described above were used for analysis, of which 93.3% (56,333 people) were classed as White Scottish. Of the minority ethnic populations, the largest group was Pakistani, with 4.2% (2,535 people), and the smallest ethnic group was African-Caribbean with 0.4% (223 people). Collectively, South Asians (Indians and Pakistanis) represented 5.6% (3,392 people) of the study population. Characteristics of people with missing/implausible data were similar to those included in the analyses (data available on request).

The median HbA1c for each ethnic group (calculated by taking the median value of all the median values for each person for the respective ethnic group) were as follows: White Scottish −55 mmol/mol (Interquartile range [IQR]: 49–66 mmol/mol), African-Carribbean −60 mmol/mol (IQR: 51–73 mmol/mol), Chinese −54 mmol/mol (IQR: 50–65 mmol/mol), Indian −60 mmol/mol (IQR: 51–73 mmol/mol) and Pakistani −62 mmol/mol (IQR: 54–76 mmol/mol). The difference in median HbA1c between each ethnic group and the White Scottish was significant for the African-Caribbean, Indian and Pakistani comparisons (p = 0.043, p<0.001 and p<0.001 respectively).

**Figure 2:**
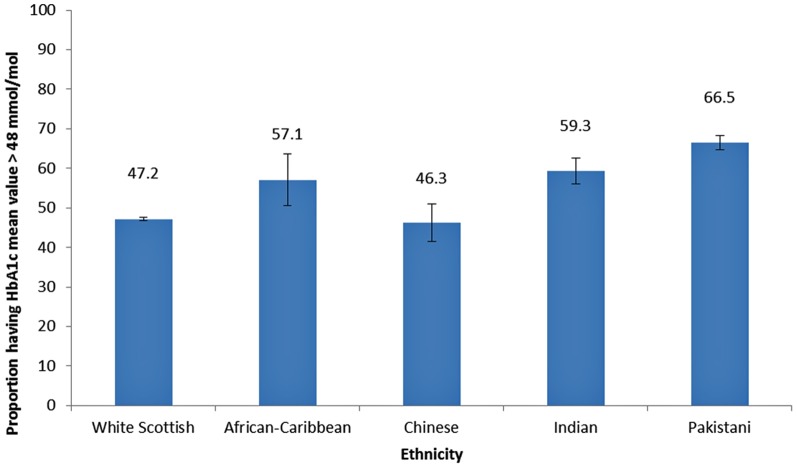
Proportion of people with Type 2 diabetes in Scotland with mean of all recorded HbA1c measurements >58 mmol/mol (7.5%) by ethnicity.

All non-white ethnic groups except for the Chinese had significantly greater (p<0.05) proportions of people with suboptimal glycaemic control compared to the White Scottish group regardless of whether mean or recent measures were used for the definition. More than half the people in each minority ethnic group had mean glycaemic control >58 mmol/mol (7.5%), with the exception of the Chinese group, of which only 46% had HbA1c >58 mmol/mol (7.5%) (Figure 2). Pakistanis had the highest proportion with suboptimal control.

The characteristics of the study population by ethnicity are shown in Table 1. The mean age of the people included in this analysis (at the time of data extraction) was 66.1 years (standard deviation 12.0 years). Among the South Asians and African-Caribbeans, there were greater proportions of people in the 31–50 years age group relative to the white Scottish group. As expected from patterns observed in the general population, distribution of socio-economic status differed by ethnic group: almost a third of the Indians were in the most affluent fifth while the African-Caribbean group had the largest proportion of any ethnic group identified as the most deprived. There were higher proportions of men than women in all ethnic groups, with the exception of the Chinese. Whilst the largest proportion of White Scottish people was in the 30.0–30.99 kg/m^2^ BMI category, in all the other ethnic groups, the most prevalent BMI group was 25–29.99 kg/m^2^.

**Table 1:** Comparison of demographic and clinical variables among ethnic groups with Type 2 DM living in Scotland (n = 56713).

	Ethnic groups, n (%)					
Variable	White Scottish	African-Caribbean	Chinese	Indian	Pakistani	
Gender^a^						
Males (n = 30,621)	28,461 (53.8)	116 (59.2)	195 (49.4)	498 (62.2)	1,351 (55.4)	
Females (n = 26,092)	24,423 (46.2)	80 (40.8)	200 (50.6)	303 (37.8)	1,086 (44.6)	
Age at diagnosis^b^						
≤30 (n = 141)	105 (0.2)	3 (1.5)	4 (1.0)	8 (1.0)	21 (0.9)	
31–50 (n = 6,124)	5,145 (9.7)	65 (33.1)	60 (15.2)	195 (24.3)	659 (27.1)	
51–70 (n = 28,125)	26,043 (49.3)	101 (51.6)	204 (51.6)	426 (53.2)	1,351 (55.4)	
>70 (n = 22,323)	21,591 (40.8)	27 (13.8)	127 (32.1)	172 (21.5)	406 (16.7)	
SES						
1 (most affluent) (n = 9,041)	8,074 (15.3)	31 (15.8)	101 (25.6)	261 (32.6)	574 (23.6)	
2 (n = 9,620)	8,905 (16.8)	21 (10.7)	76 (19.2)	179 (22.3)	439 (18.0)	
3 (n = 10,325)	9,845 (18.6)	16 (8.2)	43 (10.9)	99 (12.4)	322 (13.2)	
4 (n = 13,124)	12,202 (23.1)	37 (18.9)	72 (18.2)	140 (17.5)	673 (27.6)	
5 (most deprived) (n = 14,603)	13,858 (26.2)	91 (46.4)	103 (26.1)	122 (15.2)	429 (17.6)	
Average of all BMI values (kg/m^2^)						
<18.5 (n = 112)	103 (0.2)	0 (0.0)	1 (0.3)	2 (0.2)	6 (0.2)	
18.5–24.99 (n = 6,445)	5,664 (10.7)	31 (15.8)	158 (40.0)	199 (24.8)	393 (16.1))	
25–29.99 (n = 19,964)	18,239 (34.5)	88 (44.9)	176 (44.6)	374 (46.7)	1,087 (44.6)	
30–39.99 (n = 25,399)	24,216 (45.8)	62 (31.6)	56 (14.2)	204 (25.5)	861 (35.3)	
≥40 (n = 4,793)	4,662 (8.8)	15 (7.7)	4 (1.0)	22 (2.7)	90 (3.7)	
Duration of diabetes in years						
≤1 (n = 4,070)	3,778 (7.1)	21 (10.7)	26 (6.6)	68 (8.5)	177 (7.3)	
2–5 (n = 14,405)	13,265 (25.10	67 (34.2)	130 (32.9)	241 (30.1)	702 (28.8)	
6–10 (n = 20,617)	19,365 (36.6)	65 (33.2)	119 (30.1)	251 (31.3)	817 (33.5)	
11–15 (n = 10,296)	9,693 (18.3)	25 (12.8)	68 (17.2)	112 (14.0)	398 (16.3)	
>15 (n = 7,325)	6,783 (12.8)	18 (9.2)	52 (13.2)	129 (16.1)	343 (14.1)	
Treatment prescribed						
Oral (n = 37,266)	34,421 (65.1)	141 (71.9)	306 (77.5)	585 (73.0)	1,813 (74.4)	
Insulin (n = 1,084)	1,025 (1.9)	8 (4.1)	4 (1.0)	11 (1.4)	36 (1.5)	
Both (n = 10,573)	9,918 (18.8)	33 (16.8)	48 (12.2)	122 (15.2)	452 (18.5)	
None/Unrecorded (n = 7,790)	7,520 (14.2)	14 (7.1)	37 (9.4)	83 (10.4)	136 (5.6)	

For each minority ethnic group, differences in all variables showed statistically significant association (p<0.01) compared to the Scottish group unless stated otherwise.

^a^p = 0.13 for comparison between White Scottish and African-Caribbean; p = 0.12 for comparison between White Scottish and Pakistani; p = 0.07 for comparison between White Scottish and Chinese;

^b^at time of data extraction;

^c^mean of all available BMIs for each person.

The largest proportions of White Scottish and Pakistanis within the duration categories had type 2 diabetes for 6–10 years; whereas in the other groups the most common duration was 2–5 years. People from the four minority ethnic groups were more likely to be on oral hypoglycaemic agents compared to the White Scottish group.

### Glycaemic control

Table 2 summarises the proportion of people with optimal glycaemic control (<58 mmol/mol, 7.5% cut-off) for the variables under analysis. Males tended to have poorer control than females and younger people tended to have poorer control than the elderly. Similarly, the proportion of people with optimal glycaemic control decreased as BMI increased. Increasing deprivation was associated with decreasing proportion of people with optimal glycaemic control. As expected, measures of disease progression including treatment and duration of diabetes were associated with glycaemic control with a more marked association between treatment than duration of diabetes. Sensitivity analyses demonstrated that these associations were similar for mean HbA1c and most recent HbA1c values and when using a <53 mmol/mol (7%) cut-off to categorise glycaemic control.

**Table 2:** Proportion of people with Type 2 DM in Scotland having optimal glycaemic control (mean HbA1c cut-off 58 mmol/mol, 7.5%) for demographic and clinical variables (n = 56713).

Variables	Optimal glycaemic control, n (%)
Gender	
Males (n = 30,621)	15,622 (51.0)
Females (n = 26,092)	13,761 (52.7)
Age (years)	
≤30 (n = 141)	49 (34.8)
31–50 (n = 6,124)	2,288 (37.4)
51–70 (n = 28,125)	13,437 (47.8)
>70 (n = 22,323)	13,609 (61.0)
Mean BMI (kg/m^2^)	
<18.5 (n = 112)	76 (67.9)
18.5–24.99 (n = 6,445)	3,711(57.6)
25–29.99 (n = 19,964)	10,942 (54.8)
30–39.99 (n = 25,399)	12,533 (49.3)
≥40 (n = 4,793)	2,121 (44.3)
Treatment prescribed	
Oral (n = 37,266)	20,129 (54.0)
Insulin (n = 1,084)	315 (29.1)
Both (n = 10,573)	1,602 (15.2)
None/Unrecorded (n = 7,790)	7,337 (94.2)
Socioeconomic status (SIMD quintiles)	
1 (most affluent) (n = 9,041)	4975 (55.0)
2 (n = 9,620)	5,199 (54.0)
3 (n = 10,325)	5,400 (52.3)
4 (n = 13,124)	6,794 (51.8)
5 (most deprived) (n = 14,603)	7,015 (48.0)
Duration of diabetes	
≤1 (n = 4,070)	2,350 (57.7)
2–5 (n = 14,405)	8,735 (60.6)
6–10 (n = 20,617)	11,184 (54.2)
11–15 (n = 10,296)	4,484 (43.6)
>15 (n = 7,325)	2,630 (35.9)

All associations between each variable and proportion of optimal mean glycaemic control was statistically significant (p<0.001)

### Univariate and multivariate analyses of glycaemic control

Table 3 compares the odds ratios (ORs) for poor glycaemic control between the White Scottish group (reference group) and other ethnic groups. The crude ORs of having suboptimal glycaemic control compared to the White Scottish group were significantly higher in all the other ethnic groups except for the Chinese. Adjustment for age and sex attenuated the ORs in all ethnic groups, with the OR only remaining significant in the Indians and Pakistanis. After adjusting for potential confounding variables (age, gender, SES, BMI, treatment prescribed, and duration) ***as identified*** using backward stepwise variable selection in the sub-group of people for whom these data were available, statistically significant increased odds of suboptimal glycaemic control persisted in Pakistanis (OR 1.85, 95% CI 1.68–2.04) and Indians (1.62, 95% CI 1.38–1.89). The changes in point estimates in the different models reflect the younger age and lower BMI of ethnic minority groups compared to the white Scottish group

**Table 3:** Crude and adjusted odds ratios for suboptimal glycaemic control (mean HbA1c >58 mmol/mol, 7.5%) compared to the white Scottish population (n = 56713).

	Ethnic groups, Odds Ratio (95% CI)				
	White Scottish (reference)	African-Caribbean	Chinese	Indian	Pakistani
**Crude**	1.00	1.50 (1.13–1.98)	0.97 (0.79–1.18)	1.63 (1.42–1.88)	2.22 (2.04–2.42)
**Age and sex adjusted**	1.00	1.15 (0.87–1.54)	0.89 (0.73–1.08)	1.36 (1.18–1.58)	1.78 (1.63–1.94)
**Age-, sex- and SES-adjusted**	1.00	1.12 (0.84–1.5)	0.90 (0.74–1.10)	1.43 (1.24–1.65)	1.83 (1.68–2.00)
**Age, sex, SES and BMI adjusted**	1.00	1.17 (0.87–1.56)	0.97 (0.79–1.19)	1.51 (1.30–1.74)	1.91 (1.74–2.08)
**Fully adjusted**	1.00	1.11 (0.81–1.51)	1.01 (0.81–1.26)	1.62 (1.38–1.89)	1.85 (1.69–2.04)

Adjusted variables - sex, age socioeconomic status (SES), BMI, prescribed treatment and duration

## Discussion

The SCI-DC dataset allowed us to address different factors affecting glycaemic control in patients with type 2 DM across Scotland based on ethnicity using data collected since the introduction of the Quality Outcomes Framework in 2004 which may have been expected to reduce inequalities in glycaemic control. The other novel aspects of this work included separation of data for Indian and Pakistani populations (unlike conventional aggregation, perhaps also with Bangladeshis and Sri Lankans) in a South Asian group and presentation of data for African-Caribbean and Chinese populations with diabetes in Scotland for the first time. This separation is important since clear difference in CVD risk have been found between Indians and Pakistanis as demonstrated in QRISK2 risk calculator [16]. Distribution of age, sex and SES differed by ethnicity and were associated with differences in HbA1c suggesting that they may confound the association between ethnicity and glycaemic control. Our study showed that Indians and Pakistanis have increased odds of suboptimal glycaemic control compared to the White Scottish population after adjustment for confounding variables. We also for the first time describe data on Chinese and African-Caribbean people with diabetes living in Scotland. We also demonstrate no increased prescription of insulin in several minority ethnic groups (Indian, Pakistani and Chinese) compared to the White Scottish group, despite these minority ethnic groups having suboptimal control.

Although we did not aim to describe aetiological factors for type 2 DM, our study also showed that diabetes is accompanied by a lower BMI in ethnic minority groups when compared to the White Scottish population. These results, like several previous studies, support the concept that the association between BMI and type 2 DM risk differs between ethnic groups, with Asians (Chinese and South Asians) experiencing adverse metabolic effects at lower BMIs [7], [17].

Low socioeconomic status is known to be associated with poor health outcomes including mortality in type 2 DM in Scotland [14]. Whilst initial adjustments which included deprivation attenuated the odds of suboptimal glycaemic control in African-Caribbeans; in Indians and Pakistanis, the odds attenuated, but remained significantly higher. Recent data from London also demonstrated that whilst social deprivation had some influence on glycaemic control in South Asians and African-Caribbeans, differences remained after adjustment [12]. Also, our results suggest that Indians in particular live in less deprived areas compared to other ethnic groups in Scotland. However measurement of SES in ethnic minority groups is notoriously difficult and thus these findings should not be over-interpreted [18], [19].

It has been recognised that South Asians are less likely to receive insulin than the indigenous population in the UK, despite having poorer glycaemic control [9], [10], although more recent data may suggest that insulin usage is increasing in South Asians [12]. A relatively recent study has shown SAs were more likely to receive oral hypoglycaemic agents (AOR 2.27; CI, 1.79–2.86) but less likely to receive insulin (AOR0.54; CI, 0.42–0.69) than the white group [10]. Reasons for this possibly include increased reluctance to take up treatment [20], patients' lack of understanding of their diagnosis [21] or attitudes shown by health professionals [22].

The dataset used in this study has several strengths. It contained information for 198,622 people with type 2 diabetes, with validation of the diagnosis by using criteria to exclude people with late-onset type 1 diabetes. Even after excluding missing data, the study population included data for 56,000 people. Further, our data was nationally representative, making use of electronic data from over 99% of primary care practices in Scotland, with previous ethnicity-based studies being regional [8], or from smaller geographical locations [7], [9], [10]. While many other studies have analysed cross-sectional data, the advantage with this dataset was the availability of multiple HbA1c and BMI values for each patient, thus providing the opportunity to summarize the overall mean measures of glycaemic control and BMI for the available data (although ethnic differences were similar when the most recent values were used in a sensitivity analysis). This dataset also gave the opportunity to compare the main South-Asian ethnic groups individually, rather than collectively against the White Scottish group. Classification of ethnicity into collective groups, namely South Asians and Europeans, has been reported as a weakness by underestimating or overlooking the degree of difference between South Asian sub- groups [23]. Heterogeneity within South Asians has been reported for several other cardiovascular risk factors, including blood pressure and smoking [23]–[25] as well as for CVD risk, which seem highest in Pakistanis [16].

This study is not without its limitations. Some of the statistically significant differences reported in tables 1 and 2 may have occurred by chance as a consequence of multiple testing. Ethnicity was poorly recorded, leading to a large proportion of missing data and it is possible that the sub-group whose characteristics we have described is not representative of the whole population of people with diabetes. Information about country of birth, time since migration or general practice was not available to us. It is not clear to what extent higher HbA1c in Pakistanis and Indians reflects higher HbA1c at diagnosis of diabetes, a faster rise in HbA1c following diagnosis, under-treatment or poorer compliance or response to lifestyle interventions or pharmacological treatments, ethnic differences in prevalence of anaemia and haemoglobinopathy and all of these factors may be relevant. This study did not address differences in treatment between ethnic groups, as data were only available on recording of prescribed treatment at the time of data extraction in 2008, with no data available on drug dosage or treatment duration or validation of these data in this analysis. Differences in drug treatment may partly explain the disparities in glycaemic control. Another limitation of this analysis is that data on ethnicity was only recorded for 38% of the patients with type 2 DM, although the study population appeared to be representative of the whole cohort, as described earlier. Unfortunately we did not have the opportunity to use name recognition software to attempt to assign ethnicity before the pseudonymised research extract was performed and the approvals we have for use of the data prevent use of the identifiable data - more recently there has been an improvement in recording ethnicity data [4], with 78% of patients having their ethnicity recorded in 2011 [26]. It was not possible to validate the recording of ethnicity or other variables in the dataset. The absence of a single, uniform standard for collecting and reporting diabetes-related information and testing HbA1c is likely to have resulted in differences in the quality of the data as also the number and the frequency of HbA1c tests conducted. As we used mean values to summarise glycaemic control and other risk factors, we were unable to investigate whether ethnicity is associated with variability of HbA1c and other factors which has been found to be associated with mortality in elderly people with diabetes [27]. Finally, the small numbers of Bangladeshis in this dataset limited our ability to investigate glycaemic control in this population.

In conclusion, our nationally representative data show that Indians and, more strikingly, Pakistanis had significantly increased odds of suboptimal control than the White Scottish population both before and after adjusting for potential confounders, whereas proportions with suboptimal glycaemic control were similar to the Scottish population in Chinese or African-Caribbean groups. The role of other factors such as treatment-specific responses, physician to patient communication as way to improve glycaemic control in South Asians and the association between glycaemic control and complications of diabetes including hospital admissions are clearly relevant areas for future research.
